# Supplementary material for: Inhibition of α(1,6)fucosyltransferase: Effects on Cell Proliferation, Migration, and Adhesion in an SW480/SW620 Syngeneic Colorectal Cancer Model
Source: Int J Mol Sci. 2022 Jul 30;23(15):8463. doi: 10.3390/ijms23158463 (PMC9369121; doi:10.3390/ijms23158463)
Supplement: Supplementary file 1 [file ijms-23-08463-s001.zip › ijms-1817910-supplementary.pdf]

**Supplementary Materials**

**Supplementary Table S1.** shRNA-*FUT8* target sequences.

| shRNA ID | Symbol | Target sequence |
|---|---|---|
| TRCN0000035952 | #52 | GTCTATAATGACGGATCTATA |
| TRCN0000035953 | #53 | CCACAGATGACCCTTCTTTAT |
| TRCN00000229959 | #59 | GAACTGGTTCAGCGGAGAATA |
| TRCN00000229960 | #60 | CTTTAGATGACATCTACTATT |
| TRCN00000229961 | #61 | TACCCATGCACAGTACAATAA |

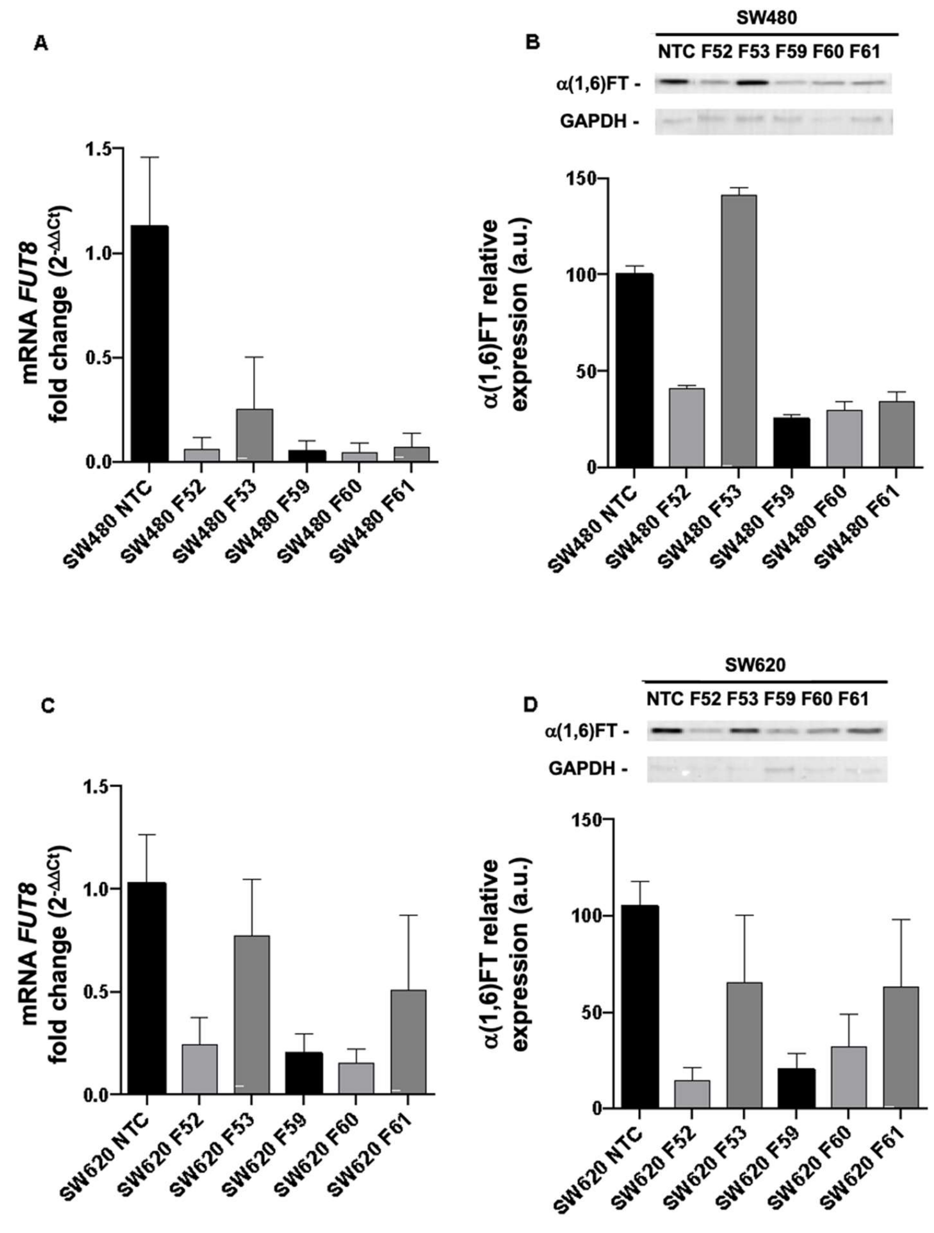


**Supplementary Figure S1.** *FUT8* mRNA and protein expression in SW480 and SW620 cell lines after shRNA-mediated *FUT8* knockdown. (**A,C**) *FUT8* mRNA levels quantified by RT-qPCR in SW480 and SW620 cells transfected with non-targeted control (NTC) and their respective *FUT8*-silenced clones F52, F53, F59, F60, and F61. Results are presented as ΔCt values between *GAPDH* and *FUT8* genes. Relative expression among cell groups was determined using $2^{-\Delta\Delta Ct}$ fold change method. Measurements were plotted as mean ± SEM. NTC clone was used as reference. (**B,D**) Western blot analysis of α(1,6)FT protein levels in SW480 and SW620 cells transfected with NTC and their respective *FUT8* knockdown clones F52, F53, F59, F60, and F61. GAPDH expression was used as loading control. SW480 intensity was equal to 100 and corresponding SW620 protein expression was referenced. Measurements were plotted as mean ± SEM. All analyses came from 2 replicates.

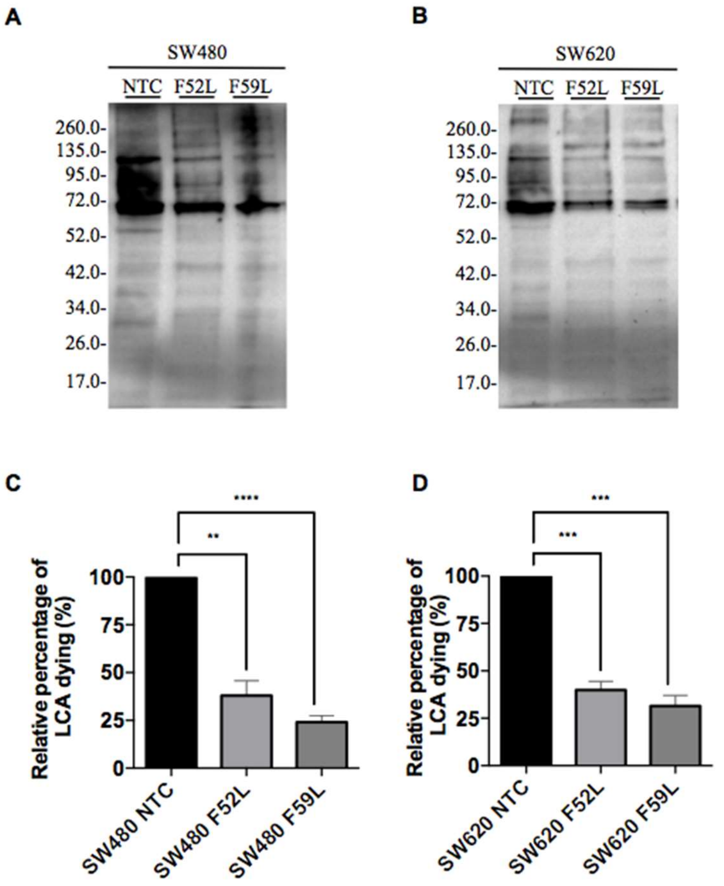


**Supplementary Figure S2.** Effect of LCA selection on core fucosylation of SW480 and SW620 cells silenced for *FUT8* gene. (**A,B**) Lectin blots of SW480 and SW620 *FUT8* knockdown clones that survived LCA selection (500 μg/mL for 72 hours). (**C,D**) LCA staining in SW480 and SW620 blots with ECL chemiluminescence was used as core fucosylation reporter. Lectin band intensity for each CRC clone was semi-quantified using R-250 Blue Coomassie dye as protein loading control. Measurements were plotted as mean ± SEM. NTC clones were used for statistical calculations. All analyses had 3 replicates. Unpaired t-test was used for statistical analyses; **$p < 0.001$; ****$p < 0.001$.

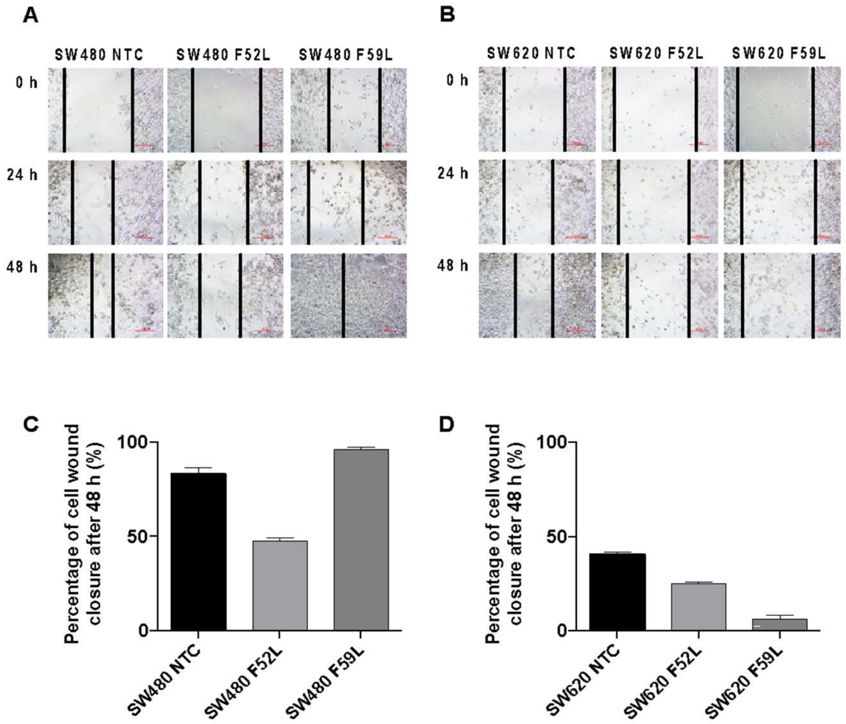


**Supplementary Figure S3.** Impact of *FUT8* knockdown on migration capacity of SW480 and SW620 CRC lines. (**A,B**) Wound closure of *FUT8*-silenced F52L and F59L clones obtained from SW480 and SW620 and of their corresponding non-targeted transfected controls (NTC). (**C,D**) Relative percentage of closure was calculated considering 0% as basal point of each cell line. Plots represent mean ± SEM of 2-3 replicates.
